# Online physical exercise and the neuropsychiatric symptoms in patients with dementia: a cross-sectional study during the COVID-19 pandemic

**DOI:** 10.1590/1980-5764-DN-2021-0079

**Published:** 2022-06-03

**Authors:** Caroline Dalla Nora, Juliana Dias de Lima, Ivan Abdalla Teixeira, Felipe de Oliveira Silva, Júlia Silva de Almeida, Fernanda Castro Monteiro, Valeska Marinho, Marcia Cristina Nascimento Dourado, Andrea Camaz Deslandes

**Affiliations:** 1 Universidade Federal do Rio de Janeiro, Instituto de Psiquiatria, Rio de Janeiro RJ, Brazil.

**Keywords:** SARS-CoV-2, Dementia, Cognitive Dysfunction, Mental Health, Exercise, SARS-CoV-2, Demência, Disfunção Cognitiva, Saúde Mental, Exercício Físico

## Abstract

Social isolation is necessary during the COVID-19 pandemic but can be harmful to mental health, especially in people with neurocognitive disorders. Although physical exercise can alleviate neuropsychiatric symptoms and improve quality of life (QoL), sedentary behavior increased during the pandemic. Online interventions can contribute to improving physical activity and mental health.

**Objective:**

The objective of this study was to compare the neuropsychiatric symptoms and QoL of older adults with neurocognitive disorders who participated in an online physical exercise program with sedentary patients during the COVID-19 pandemic.

**Methods:**

In this cross-sectional study, 25 older patients with neurocognitive disorders (control group=11; online exercise group=14) were evaluated based on Neuropsychiatric Inventory (NPI) and the Quality of Life in Alzheimer’s Disease (QoL-AD) scale.

**Results:**

There were differences between the two groups in the total NPI (U=36.50, p=0.025) and the nighttime behavior disturbances item (U=38.00, p=0.033), both with large effect sizes (ES=-1.03, 95% confidence interval [CI]:-1.83 to -0.16 and ES=-1.06, 95%CI -1.86 to -0.19, respectively). In terms of QoL-AD, a difference was identified only in the memory subitem (U=20.00, p=0.005), with a large ES (1.59, 95%CI 0.59-2.48).

**Conclusions:**

Older adults with neurocognitive disorders who participated in an online physical exercise program, during the COVID-19 pandemic, showed fewer neuropsychiatric total symptoms, fewer nighttime disturbances episodes, and better subjective memory, compared to their physically inactive counterparts. Randomized controlled trials should be performed to better understand the effect of physical exercise in neuropsychiatric symptoms in dementia patients during periods of social isolation.

## RESUMO.

O isolamento social é necessário na pandemia de COVID-19, mas pode impactar a saúde mental, especialmente em idosos com demência, dada a alta prevalência de sintomas neuropsiquiátricos. Apesar da prática de exercícios físicos contribuir para a redução desses sintomas e a melhora da qualidade de vida, houve um aumento de comportamento sedentário durante a pandemia.

**Objetivo:**

Comparar os sintomas neuropsiquiátricos e a qualidade de vida de idosos com distúrbios neurocognitivos que participaram de um programa de exercícios físicos *online* voltado a pacientes sedentários durante a pandemia de COVID-19.

**Métodos:**

Neste estudo de corte transversal, 25 idosos diagnosticados com transtorno neurocognitivo (controle=11; exercícios *online*=14) foram avaliados por meio do inventário neuropsiquiátrico (INP) e da escala de qualidade de vida na doença de Alzheimer (QV-DA).

**Resultados:**

Observou-se diferença entre os grupos no INP total (U=36,50, p=0,025), com tamanho de efeito grande (effect size [ES]=-1,03, intervalo de confiança - IC95% -1,83 a -0,16), e no subdomínio sono (U=38,00, p=0,033), com tamanho de efeito grande (ES=-1,06, IC95% -1,86 a -0,19), favoráveis ao grupo fisicamente ativo. Na QV-DA, houve diferença entre os grupos apenas no subitem memória (U=20,00, p=0,005), com tamanho de efeito grande (ES=1,59, IC95% 0,59 a 2,48), não houve diferença na pontuação total (U=45,5, p=0,277).

**Conclusões:**

Idosos submetidos a rotina de exercícios físicos com supervisão *online* na pandemia de COVID-19 apresentam menos sintomas neuropsiquiátricos, melhor qualidade de sono e memória quando comparados aos fisicamente inativos. Estudos randomizados controlados devem ser feitos para a melhor compreensão dos efeitos do exercício físico nos sintomas neuropsiquiátricos de pacientes com demência durante períodos de isolamento social.

## INTRODUCTION

Government recommendations for social distancing were implemented during the COVID-19 pandemic to protect the public, particularly older adults at higher risk of serious complications or death due to ­SARS-CoV-2 infection^1^ ^,^ ^2^. Although the primary purpose of social isolation is to reduce infection rates, studies have also shown the negative impacts of prolonged quarantine periods on aspects of mental health, such as increased anxiety (*Odds Ratio* [OR]=2.92, 95% confidence interval [CI] 2.43-3.51) and depressive symptoms (OR=4.55, 95%CI 3.82-5.41) in the general population^3^. In particular, studies in elderly people with neurocognitive disorders have revealed positive associations between social isolation and worsening of mental health during the COVID-19 pandemic^4^ ^,^ ^5^. A multicenter national survey conducted in 89 centers for cognitive disorders and dementia in Italy investigated the impact of quarantine after 45 days of social isolation on 4,913 patients with dementia. This study revealed a worsening of cognition in 55% of the sample, as well as neuropsychiatric symptoms of irritability (40%), apathy (35%), and agitation (31%)^5^. A review study on the experience of people with neurocognitive disorders in dealing with the COVID-19 pandemic indicated a worsening of neuropsychiatric and cognitive symptoms as well as an increase in caregiver burden during this period^6^.

Neuropsychiatric symptoms have an impact on a patient’s quality of life (QoL) and a caregiver’s burden and well-being, contributing to early institutionalization^7^ ^,^ ^8^ ^,^ ^9^. Behavioral and psychological symptoms of dementia are more associated with QoL than cognition and functionality^10^. Recently, Dourado et al.^11^ verified that mood, functionality, and awareness of morbidity are predictors of QoL in patients with Alzheimer’s disease (AD). In acute situations, psychotropic drugs can be used to treat symptoms that endanger the safety of the patient or caregiver^12^. The administration of this treatment should be performed for the shortest possible time, since these drugs are associated with potential side effects. Antipsychotics are associated with an increased risk of sudden death and cardiovascular events^13^, and the use of selective serotonin reuptake inhibitors and venlafaxine as antidepressants is associated with an increased risk of hyponatremia^14^. Exercises, beyond effectiveness in multidomain in AD, showed cost-effectiveness to behavioral and psychological symptoms^15^ ^,^ ^16^. Therefore, if possible, neuropsychiatric symptoms should be managed through non-pharmacological measures^7^.

Studies have shown that therapeutic activities accompanied by music^18^ and physical exercise^19^ ^,^ ^20^ have beneficial effects on neuropsychiatric symptoms in the elderly. In particular, studies have indicated the positive effects of physical exercise on neuropsychiatric and depressive symptoms and QoL in people with cognitive impairment^20^. Physical exercise improves the QoL in elderly people with neurocognitive disorders^21^. A study conducted in patients with dementia in a long-term institution showed that participation in combined exercises was associated with an improvement in QoL compared to the control group^22^. In the general population, it is known that strategies incorporating physical exercise have a favorable effect on mental health since a positive correlation is observed between the level of habitual physical activity and the feeling of mental well-being during quarantine^23^ ^,^ ^24^ combined with fewer symptoms of depression and anxiety^25^.

In this study, we investigated the neuropsychiatric symptoms and QoL in elderly people with neurocognitive disorders during the social isolation period caused by the COVID-19 pandemic, to compare the differences between physically inactive patients with those who participated in a remotely supervised physical exercise program.

## METHODS

### Study design and participants

This cross-sectional case-control study was conducted between August and November 2020. All subjects were outpatients being followed up at the Alzheimer’s Disease Center of the Institute of Psychiatry of the Universidade Federal do Rio de Janeiro (Brazil). The diagnosis was previously made by medical staff based on a structured clinical interview according to the Diagnostic and Statistical Manual of Mental Disorders (DSM-V)^26^ and the criteria of Petersen^27^.

Elderly men and women aged over 65 years with a clinical diagnosis of a major neurocognitive disorder, such as AD (n=14), vascular disease (n=1), Lewy bodies (n=1), frontotemporal dementia (n=1) or unspecified (n=5), or mild neurocognitive disorder (n=2), were invited to participate with their caregivers. The exclusion criteria were as follows: history of severe heart disease, acute or chronic musculoskeletal injuries that prevent exercise, severe cognitive decline, and other mental disorders.

A priori sample calculation was performed using G*Power software, version 3.1.9.2. It was found an effect size (ES) of 1.46, a power of 0.95 (95%), and a type error α=0.05 (5%), which suggested a sample size of 22 individuals (11 in each group). The ES calculation was based on means and standard deviations (SDs) suggested by Stella et al.^28^ (control: M=43.3; SD=18.4 and physical activity group: M=16.9; SD=17.6), which evaluate the effect of a physical activity program on the neuropsychiatric symptoms of older adults with AD^26^.

The exercise group comprised patients who already exercised before the pandemic and maintained the routine exercises throughout a remotely supervised physical exercise program. The online tool used to provide the program was Zoom’s platform. The remotely supervised physical exercise routine consisted of a structured program of aerobic (stationary and varied walks), strength (standing up and sitting down, elbow flexion), coordination, flexibility and balance (one-foot support, plantar flexion) activities, as well as cognitive engagement dual-tasks (performing two concomitant tasks, motor/cognitive, as answering questions during the movement, trail guided by letters or colors) lasting 60 min. The activities were performed twice a week for at least 3 months in online groups of a maximum of 10 patients and their caregivers, always supervised by a physical education professional. Accessories including a plastic bottle filled with water or earth, towels, and cushions were adapted to meet the needs of each patient. The activities were of mild to moderate intensity and modified according to the capacity of each individual. The control group comprised other patients who were in outpatient follow-up and did not perform any type of exercise during the evaluation period.

This study was approved by the Ethics Committee (CAAE: 35449820.0.0000.5263), and all patients provided informed consent to participate via an online form before the beginning of the evaluations.

### Procedures and measures

Evaluation of global cognitive capacity was performed at the time of the patient’s initial evaluation by medical staff at the Alzheimer’s Disease Center using the Mini-Mental State Examination^29^, the Verbal Fluency Test^30^, and the Clinical Dementia Rating^31^. This information was obtained through accessing medical records. The patient was interviewed through telephone contact via the main caregiver. The assessment included an evaluation of anamnesis and a structured questionnaire designed to collect sociodemographic data (e.g., sex, age, education, and marital status) and details of the patient’s psychological symptoms and QoL. The evaluation was performed 6 months after the onset of quarantine and 3 months after starting the online exercise program.

Neuropsychiatric symptoms were evaluated using the Neuropsychiatric Inventory (NPI)^32^, which comprises a questionnaire delivered by the caregiver, consisting of 12 domains (i.e., hallucinations, delusions, agitation, depression, anxiety, euphoria, apathy, disinhibition, irritability, aberrant motor behavior, and sleep and eating disorders), each evaluated in terms of frequency and intensity, with scores ranging from 0 to 144 points^33^.

The QoL of patients was assessed according to the Quality of Life in Alzheimer’s Disease (QoL-AD) scale based on the answers provided by the main caregiver. The QoL-AD scale contains 13 items (i.e., physical health, energy, mood, living situation, memory, family, marriage, friends, ability to do chores, ability to do things for fun, self, money, and life as a whole), and scores ranging from 13 to 52 are directly proportional to a better QoL^34^ ^,^ ^35^.

### Statistical analysis

A descriptive analysis of the demographic data was conducted. The Kolmogorov-Smirnov and Levene’s tests were applied to verify normal distribution and homoscedasticity of the data, respectively. The demographic characteristics and NPI and QoL scores were compared between groups (remotely supervised physical exercise×control group) using Student’s *t*-test (parametric variables), the Mann-Whitney U tests (nonparametric variables), and χ^2^ test (categorical variables). Cohen’s coefficient was used to assess ES magnitude as small (>0.20), moderate (>0.50), or large (>0.80), with 95%CI^36^. All statistical analyses were performed using SPSS^®^ version 26.0 and GraphPad^®^ version 5.01. The value of p≤0.05 was considered statistically significant.

## RESULTS

The final sample consisted of 25 patients: 11 patients in the control group and 14 patients in the exercise group (remotely supervised physical exercise). The details of the sociodemographic characteristics, cognitive characteristics, neuropsychiatric symptoms, and QoL-AD of the study participants are presented in Table 1.

**Table 1.** Demographic and clinical characteristics by groups.

		Total (n=25)	Control group (n=11)	Physically active (n=14)	F/χ^2^ (p-value)
Age (years)^a^		78.0 (4.0)	77.3 (7.5)	78.6 (7.9)	-0.40 (0.69)
Disease duration (years)^a^		6.0 (4.0)	6.5 (3.4)	5.7 (4.3)	0.43 (0.67)
Sex	Male (%)	52	45.5	57.1	0.33 (0.56)
	Female (%)	48	54.5	42.9	
Marital status, n (%)	Married	12 (50)	5 (50)	7 (50)	0.17 (0.91)
	Divorced	4 (16.7)	3 (30)	5 (35.7)	
	Widower	8 (33.3)	2 (20)	2 (14.3)	
Education (%)	0-4 years	24.0	27.3	21.4	5.21 (0.15)
	5-9 years	32.0	27.3	35.7	
	10-12 years	32.0	18.2	42.9	
	>12 years	12.0	27.3	0.0	
MMSE (score)^a^		22.2 (3.6)	20.7 (3.7)	23. (3.3)	-1.85 (0.07)
Verbal fluency (score)^a^		14.2 (7.4)	14.6 (8.8)	14.0 (6.4)	0.20 (0.83)
CDT (score)^b^		2.0 (0-5)	1.0 (0-3)	2.5 (0-5)	35.50 (0.04)*
CDR (score)^b^		1.1 (0-2)	1.2 (0-2)	1.0 (0.5-2)	57.00 (0.53)
NPI total (score)^b^		15.0 (0-42)	22.0 (0-42)	4.5 (0-25)	36.50 (0.02)*
QoL total (score)^b^		31.0 (15-39)	29.0 (15-39)	32.5 (22-37)	45.50 (0.26)

MMSE: Mini-Mental State Examination; CDT: Clock Drawing Test; CDR: Clinical Dementia Rating; NPI: Neuropsychiatric Inventory; QoL: quality of life; *p<0.05; ^a^Parametric (mean and standard deviation); ^b^Non-parametric (median, minimum, and maximum).

The total NPI score in the exercise group (median=4.5, range=0-25) was lower than that in the control group (median=22.0, range=0-42), indicating that elderly patients with neurocognitive disorders who participated in the online supervised exercise program had significantly fewer neuropsychiatric symptoms (U=36.50, p=0.025). In the individual evaluation of the NPI scale items, there was a statistically significant difference between the groups only in the nighttime behavior disturbances item (U=38.00, p=0.033), showing that those who participated in the online supervised exercise had better sleep quality than those in the inactive group. The Mann-Whitney U-test analysis revealed that there were no significant differences between the groups in terms of the other NPI scale items.

**Figure 1.**
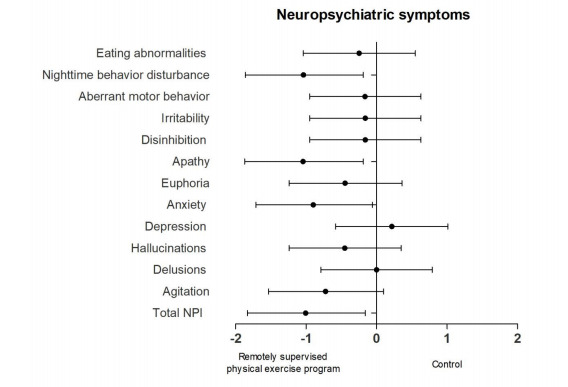
Effect sizes of neuropsychiatric symptoms by subdomain.

The NPI ES magnitude analyses (Figure 1) revealed large effects between exercise and control group on the total NPI total scores (ES=-1.03, 95%CI -1.83 to -0.16), nighttime behavior disturbances (ES=-1.06, 95%CI -1.86 to -0.19), anxiety (ES=-1.06, 95%CI -1.71 to -0.06), and apathy (ES=-1.07, 95%CI -1.87 to -0.19). Moreover, a moderate ES was observed for agitation (ES=-0.75, 95%CI -1.53-0.1). Furthermore, we observed a small but favorable effect on delusions (ES=0.0, 95%CI -0.79-0.79), hallucinations (ES=-0.46, 95%CI -1.24-0.35), euphoria (ES=-0.46, 95%CI -1.24-0.36), disinhibition (ES=-0.16, 95%CI -0.95-0.63), irritability (ES=-0.16, 95%CI -0.95-0.63), aberrant motor behavior (ES=-0.17, 95%CI -0.95-0.63), and eating abnormalities (ES=-0.25, 95%CI -1.04-0.55). The only subitem for which a worst NPI score was detected in the exercise group was depression, although the ES was small (ES=0.22, 95%CI -0.58 to -1.01).

Negative results indicate fewer neuropsychiatric symptoms compared with the control group.

**Figure 2.**
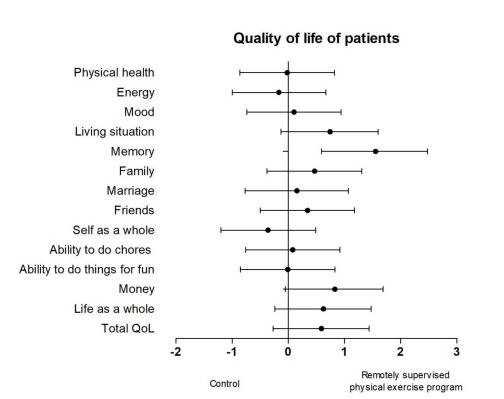
Quality of life effect sizes by subdomain.

Regarding patient’s QoL (Figure 2), there was a significant difference between groups only in the memory subitem (U=20.00, p=0.005), with a large ES (1.59, 95%CI 0.59-2.48), showing that caregivers evaluate better domain memory of people who performed physical exercise. There were no significant differences between the two groups in terms of the total QoL-AD (U=45.5, p=0.277) and other subitems.

The ES analysis revealed a moderate improvement in the total QoL-AD (ES=0.6, 95%CI -0.27 to 1.44), life as a whole (ES=0.64, 95%CI -0.23 to 1.47), and living situation (ES=0.76, 95%CI -0.13 to 1.60) subitems in the exercise group compared with the control group. The large ES in the money subitem (ES=0.85, 95%CI -0.05 to 1.69) in the exercise group indicated fewer financial concerns as assessed by the caregivers in the exercise group compared with the control group. In addition, the small ES in the family (ES=0.48, 95%CI -0.38−1.31) and friends (ES=0.35, 95%CI -0.5−1.18) subitems revealed greater satisfaction in terms of interactions with family and friends in the exercise group compared with the control group. There was also a small effect on the self subitem of the QoL-AD scale (ES=-0.3, 95%CI 1.20-0.49). However, exercise had only negligible effects on physical health, marriage, mood, ability to do chores, and ability to do things for fun subitems (ES<0.20) compared with the control group.

## DISCUSSION

In this study, we investigated the neuropsychiatric symptoms and QoL of patients with neurocognitive disorders who maintained a routine of remotely supervised physical exercise during the COVID-19 pandemic compared to those who were physically inactive. We showed that people with neurocognitive disorders who maintained a physical exercise routine during this period presented significantly fewer neuropsychiatric symptoms and better sleep than those who did not remain physically active. However, there was no difference in QoL between groups.

Social isolation caused by the COVID-19 pandemic has been indicated as a factor in increasing neuropsychiatric symptoms^37^. Lara et al. reported a significant increase in the total NPI score (p=0.028) in elderly people with mild cognitive impairment (MCI) and AD after 5 weeks of lockdown in Spain. Anxiety and apathy were the most frequently reported symptoms in the MCI group, while the AD group reported apathy and agitation. Recent studies have shown a relationship between exercise and mental well-being during the period of isolation during the COVID-19 pandemic^23^ ^,^ ^24^ ^,^ ^25^. A Brazilian study showed that those who practiced remotely supervised exercise presented fewer depressive symptoms than those who did not practice physical activity^38^. However, patients with dementia were not included in these studies. The results are in agreement with previous studies showing better neuropsychiatric symptoms in patients with dementia who participated in an exercise program^20^ ^,^ ^39^ ^,^ ^40^. In a recent meta-analysis, Dauwan et al.^41^ also observed a positive effect of physical exercise on depressive symptoms in patients with AD. The results reported in this study highlight the value of remotely supervised physical exercise as a possible alternative intervention to promote mental health in patients with neurocognitive disorders during the COVID-19 pandemic.

Sleep was the only subdomain among the NPI items for which a significant difference was identified between the exercise and control groups (p=0.03), with a large effect detected for the remotely supervised physical exercise group (ES=-1.06). This finding follows the report suggested by McCurry et al.^42^, in which sleep improvement was observed in patients with dementia following a combined intervention of sleep hygiene, exposure to light, and walking. In addition, multimodal exercise was found to attenuate sleep disturbance in AD patients^43^. The potential mechanisms by which sleep disturbance is alleviated by the exercise program include changes in core body temperature, the release of neurotransmitters that regulate sleep, increased energy consumption, changes in heart rate variability and autonomic function, and reduced inflammation^44^. As expected, we found a large ES for the anxiety and apathy subdomains, showing that people with neurocognitive disorders who participated in the online physical exercise program had fewer symptoms than those in the control group. Among the possible mechanisms associated with the anxiolytic and antidepressant effects of physical exercise, it is expected an increase in neurotransmitters and trophic factors, neurogenesis, and angiogenesis, as well as an increased activation of the opioid and endocannabinoid systems^21^.

In this study, we found that there was no difference in the total QoL-AD scores of the exercise and control groups. However, the scores in the exercise group were similar to those observed in previous studies of the QoL of people with neurocognitive disorders before the COVID-19 pandemic^11^ ^,^ ^34^. The relatively low QoL-AD scores in the control group may be associated with the effects of the pandemic and social isolation, which corroborate the findings of reduced QoL in cognitively healthy elderly individuals and those with neurocognitive disorders^45^ ^,^ ^46^. Moreover, reports on the effects of physical exercise on the QoL of people with neurocognitive disorders are inconsistent^47^, which may be due to the multifactorial characteristics of the evaluation or variability in the instruments used. In general, studies have demonstrated the benefits of exercise on the QoL of elderly who are cognitively healthy^48^ and those with depression^47^ ^,^ ^49^, as well as people with neurocognitive disorders, even dementia types^20^ ^,^ ^21^ ^,^ ^41^ ^,^ ^50^. However, the evidence is scarce and the level of evidence is low. In our QoL evaluation, memory was the only subitem for which significant difference was detected between the two groups, with a large ES identified in the group of patients who participated in the online exercise program.

This study has some limitations that need to be considered, such as the cross-sectional design and the small number of subjects. Moreover, the participants were not evaluated before and after the intervention; thus, a causal role for the intervention cannot be inferred from our data. Therefore, it is not possible to infer that those who had milder symptoms previously could be in the exercise group because they are more predisposed to perform exercises. In addition to the limitations of this study, there are barriers to implementing online interventions in dementia patients, namely, the presence of the caregiver, caregiver’s support by the teacher, interest in doing online activities, and the difficulty of digital inclusion.

Randomized controlled trials are required to further clarify the potential benefits of a remotely supervised exercise program in people with neurocognitive disorders.

Elderly people with neurocognitive disorders who participated in a remotely supervised program of physical activities had significantly better sleep quality, subjective memory, and fewer neuropsychiatric symptoms. This type of intervention seems to be a feasible option for reducing sedentary behavior and improving behavioral symptoms in people with neurocognitive disorders during the COVID-19 pandemic. However, randomized controlled trials should be performed to better understand the effect of physical exercise in dementia patients during the periods of social isolation.
